# Annotations

**Published:** 1896-08-29

**Authors:** 


### ANNOTATIONS.

### The Drink Mortality at Liverpool.

Reading through the annual report for 1895 of Dr. Hope, Medical Officer of Health for Liverpool, the attention was somewhat painfully arrested by several facts connected with the bill of mortality for the year. Among the rest, the report stated that "inquests were held upon the bodies of 110 persons whose deaths were directly caused by excessive drinking, and upon 86 whose deaths were accelerated by excessive drinking —a total of 196 persons in one year in the city of Liverpool who have been proved to have died from drink. When it is considered how many have died unnatural deaths, caused or accelerated by drink, we have the striking circumstance that at least one in every eighty-five persons who died in Liverpool last year was self-murdered by indulgence in a vile and loathsome habit. It would be well if some ardent abstainer in the medical profession, who could read between the lines of medical certificates, would investigate the facts as regards the total deaths in Liverpool which were directly or indirectly caused by drink last year; and better still, if a comprehensive inquiry could be made which would bring out all the facts of this kind accurately for the whole kingdom. If this were done in so honest and sober a way as to command confidence we venture to think that an argument would be produced for total abstinence, or at least for very moderate drinking, which would convince more than any other set of facts made public in recent years.

### Diphtheria in Soil, in Cows, and in Human Beings.

A discussion took place at the Carlisle meeting of the British Medical Association on the subject of diphtheria, but it cannot be said that much was advanced which was either definite or novel. One fact, however, was put forward by Dr. Gordon Sharp, as the result of his investigations into the relation between the soil and the organism of diphtheria which, if, in the hands of other investigators, it should receive confirmation, is likely to be of considerable importance in elucidating the etiology of the disease. From two specimens of soil, each taken from a spot at some distance away from human habitations, he cultivated bacilli which were indistinguishable from those of diphtheria. In considering the bearing of such an observation one should remember that it is by no means probable that the relation between the saprophytic and the pathogenic manifestations of the bacillus is in any way a near or direct one. It is conceivable that the damp walls or floor of a cottage might become the nidus from which case after case might be infected; but in view of our other knowledge, if on further investigation the bacillus should at all commonly be found in country soil, it would seem most reasonable to regard soil diphtheria and cow diphtheria as having direct relationship with each other, much as the anthrax of the soil and that of the animals which feed upon its herbage are related; and to look upon diphtheria in man as a mere accidental development of the disease, owing to his bad habit of drinking the uncooked milk of diseased cows, much as malignant pustule and woolsorter's disease in man are accidental developments of anthrax owing to his handling diseased hides or breathing the dust from the fleeces of diseased sheep. Of course such a theory would admit fully the spread of diphtheria by infection from child to child, just as it would the possibility of a careless surgeon infecting himself with charbon, but it would also tend in some degree to explain that very interesting phenomenon, the seasonal prevalence of diphtheria. We would by no means encourage the building of pretty theories, but facts regarding diphtheria are accumulating so rapidly that we may expect before long to possess a much more complete conception of its etiology than we have at present, and there is much to suggest that when that more perfect knowledge comes its teaching will be that if we would avoid sharing with animals their diseases, including diphtheria and scarlet fever, we must cook all animal products which we use as food.

### Night Shelters and What We Pay for Them.

Dr. Waldo and Dr. Walsh have again and again pointed out the evils both social and sanitary which are connected with the existence of cheap night shelters which are outside the control of the police and of the sanitary authorities. In bringing the matter before the British Medical Association at Carlisle, however, they have attacked the question from another point of view, and have shown what an expense the existence of the shelters entails upon the ratepayers of the districts in which they are planted. The argument they advance is that the Blackfriars Shelter, for example, attracts to St. Saviour's Union a flock of outside vagrants, and that this importation concerns all who contribute to the rates. Let us suppose, they say, that a vagrant has wandered up to London. He hears that for a penny he can sleep in the Blackfriars Shelter. He sleeps there for one night, and, not being able to find the requisite penny for another night, he throws himself on the Union of St. Saviour's. Unless the guardians are able to discover his original place of settlement they are thenceforth saddled with his maintenance. As an able-bodied man, he is kept in the workhouse; if ill, he has to be tended in the infirmary; if insane, he is put in an asylum; if he dies, he must be buried; all at the cost of the ratepayers. These responsibilities have again and again been thrust upon the guardians as the result of one or more nights spent in the Blackfriars Shelter, and they find that the direct cost to the Union for paupers who came from the shelter in 1895 amounted to £837, not including that of the "casuals." We have several times urged the sanitary dangers which attach to the uncontrolled aggregation of such numbers of destitute persons as night after night are drawn to these semi-philanthropic shelters, and to the great anomaly which is presented by their exemption from the operations of the Common Lodging Houses Acts. It would appear that the economic side of the question is no less important if, as Dr. Waldo shows, the shelters attract a poor, shiftless class of the community to what are already the most poverty-stricken districts of London, thus throwing upon them an altogether unfair burden. After the reading of Dr. Waldo's paper a resolution was unanimously passed, "That it is desirable that all public night shelters should be forthwith included within the purview of the Common Lodging Houses Acts."

